# The metabolic profiles of different fiber type populations under the emergence of the slow component of oxygen uptake

**DOI:** 10.1186/s12576-020-00754-1

**Published:** 2020-05-28

**Authors:** Sonia Conde Alonso, Trishan Gajanand, Joyce S. Ramos, Jean-Philippe Antonietti, Fabio Borrani

**Affiliations:** 1 Institute of Sport Sciences, University of Lausanne, Lausanne, Switzerland; 2 School of Human Movement and Nutrition Sciences, The University of Queensland, St Lucia, QLD Australia; 3 SHAPE Research Centre, Exercise Science and Clinical Exercise Physiology, College of Nursing and Health Sciences, Flinders University, Bedford Park, SA 5042 Australia; 4 Institute of Psychology, University of Lausanne, Lausanne, Switzerland; 5 Department of Exercise Sciences, Faculty of Science, University of Auckland, Auckland, New Zealand

**Keywords:** Oxygen consumption kinetics, Slow component, Muscle fatigue, Muscle fibers’ metabolic properties

## Abstract

To investigate the influence of different metabolic muscle fiber profiles on the emergence of the slow component of oxygen uptake (${\dot{\text{V}}\text{O}}_{2}$_SC_), 12 habitually active males completed four sessions of different combinations of work-to-work transition exercises up to severe intensity. Each transition was modeled to analyze the different kinetic parameters. Using a new approach, combining Henneman’s principle and superposition principle, a reconstructed kinetics was built by temporally aligning the start of each new transition and summing them. The primary phase time constant significantly slowed and the gain at the end (GainEnd) significantly increased when transitions started from a higher intensity (*p* < 0.001). Kinetic parameters from the reconstructed curve (${\dot{\text{V}}\text{O}}_{2} {\text{baseline}}$, time delay of primary phase, ${\dot{\text{V}}\text{O}}_{2}$End and GainEnd) were not significantly different from one transition to severe exercise. These results suggest that the appearance of the ${\dot{\text{V}}\text{O}}_{2}$_SC_ is at least related to, if not the result of, the different metabolic properties of muscle fibers.

## Background

The fundamental response of muscle oxygen consumption (${\dot{\text{V}}\text{O}}_{2}$) kinetics, during moderate transition, may closely reflect the kinetics of ${\dot{\text{V}}\text{O}}_{2}$ in the contracting muscles [1]. At a constant work rate exceeding the gas exchange threshold (GET), this response is characterized by a delayed-onset of the new metabolic requirements, defined as the ‘${\dot{\text{V}}\text{O}}_{2}$ slow component’ (${\dot{\text{V}}\text{O}}_{2}$_SC_), elevating ${\dot{\text{V}}\text{O}}_{2}$ above the ‘steady-state’ value predicted for this work rate [2–4]. This excess ${\dot{\text{V}}\text{O}}_{2}$ is a reflection of a loss of muscle efficiency [5]. To date, the putative mechanisms of ${\dot{\text{V}}\text{O}}_{2}$_SC_ are poorly understood, but several hypotheses have been proposed. Among these is the potential influence of the different metabolic response profiles of different fiber type populations during the development of the ${\dot{\text{V}}\text{O}}_{2}$_SC_. Indeed, it has been shown that the mammalian skeletal muscle is composed of different cell populations, with different metabolic and mechanical characteristics, mitochondrial content, and contractile proteins [6]. Kushmerick et al. [7] compared the ${\dot{\text{V}}\text{O}}_{2}$ of the two different muscle fiber types, demonstrating that the mechanism of control of cellular respiration is quantitatively and qualitatively different in fast and slow muscle fibers. Also, Stienen et al. [8] showed in their study using single muscle human fibers during isometric contraction, that the ATP consumption depends on the myosin isoform composition. In specific, the ATP consumption in fast IIb fibers was fourfold larger than in slow type I. In addition, it has been shown in animals that respiration of mitochondria [9], the mitochondrial volume density [10] and the mitochondrial rate of O_2_ consumption [11] are greater in type I compared with type II muscle fibers. These differences between the slow and fast switch motor units may have an impact on the kinetics of mitochondrial oxidative phosphorylation during exercise above the gas exchange threshold (GET) and thus, contribute to the appearance of the ${\dot{\text{V}}\text{O}}_{2}$_SC_.

The size principle [12] posits that skeletal muscle fibers are recruited in a hierarchical manner during exercise according to intensity. To manipulate motor unit recruitment and reveal the metabolic response profiles of different fiber type populations, “work-to-work” step exercise has been used [13–15]. For instance, transitions between low work rate intensities would be expected to solicit the recruitment of muscle fibers that are positioned lower in the recruitment hierarchy (i.e., slow type fibers), whereas a transition between high work rate intensities would be expected to involve the recruitment of muscle fibers positioned higher in the recruitment hierarchy (i.e., fast type fibers) [16]. Thus, it should be possible to distinguish the effect of the recruitment of new motor units residing higher in the recruitment hierarchy during the ${\dot{\text{V}}\text{O}}_{2}$ kinetics while completing transitions between different exercise intensities (moderate, heavy and severe). In addition, because each fiber that contributes to tension development is as a unique system unto itself, and because the pulmonary ${\dot{\text{V}}\text{O}}_{2}$ signal homogenizes any oxidative response diversity within the activated pool of motor unit, the principle of superposition might be applied.

Therefore, in accordance with the Henneman and the superposition principle, and considering that the appearance of the ${\dot{\text{V}}\text{O}}_{2}$_SC_ is due to the difference in mitochondrial oxidative phosphorylation kinetics between fibers’ types, the differences in ${\dot{\text{V}}\text{O}}_{2}$ kinetics between a single work transition and a work-to-work transition of an equal final power may be due to the temporally shift of MU activation residing higher in the recruitment hierarchy. In keeping with this, temporally aligning the beginning of each new transition [activation of new fibers positioned higher in the recruitment hierarchy (Henneman principle)] and summing them (superposition principle) to form new reconstructed kinetics should not give a different ${\dot{\text{V}}\text{O}}_{2}$ kinetics with that measured in a simple transition to equal final power.

The purpose of this study was to add novel evidence to the debate of the origins of the ${\dot{\text{V}}\text{O}}_{2}$_SC_, specifically, if the different metabolic response profiles of different fiber type populations are one of the culprits in the development of the ${\dot{\text{V}}\text{O}}_{2}$_SC_.

The hypotheses were

The time constant (τ) would be significantly smaller between low-intensity work rate transitions compared with transitions between high work rate intensities.

The reconstructed ${\dot{\text{V}}\text{O}}_{2}$ kinetics from multiple transitions would, in fact, have an identical kinetic to a simple transition at the same final intensity.

## Methods

Twelve healthy habitually active males aged 18–50 years (mean ± SD: age 24.33 ± 0.72 years, height 178.41 ± 7.76 cm, weight 76.31 ± 11.62 kg) were recruited to participate in this study. Participants were excluded if they, or their family, suffered from any heart or cardiovascular condition, bleeding disorder, or were taking prescribed medication. Participants were instructed to refrain from training and other vigorous physical activities, alcohol consumption, caffeine intake and tobacco for a minimum of 24 h before experiments. Participants were advised to arrive at the laboratory in a rested, fully hydrated, and at least 3 h postprandial state. The research protocol was accepted by local Human Participants Ethics Committee, and completed according with the seventh Declaration of Helsinki (2013). Prior to participation in the study, the protocol and possible risks involved were explained to all participants before written informed consent was collected. All participants were advised of their right to withdraw from the study at any time without prejudice.

The experimentation required five visits to the laboratory. The tests included a first session of a ramp incremental test on cycle ergometer (Velotron racemate Inc Spearfish, USA) to assess GET and peak oxygen uptake (${\dot{\text{V}}\text{O}}_{2{\text{peak}}}$). The ergometer seat and handlebars were adjusted for comfort, and the measurements were recorded to reproduce a consistent setup for the subsequent tests. On subsequent days, participants completed an additional four sessions with various combinations of work-to-work transitions across a wider range of square-wave exercises. Heart rate (RS800, Polar, Finland) and pulmonary gas exchange were continuously measured using a computerized system (Metamax 3B, Cortex GmbH, Leipzig, Germany) during all sessions. Testing took place at a similar time of the day (± 2 h), conducted in a temperature-controlled laboratory (maintained at 18 ± 1 °C).

During the ramp incremental test, participants rested for 3 min on the cycle ergometer before cycling for 6 min with a load of 60 W at a comfortable self-selected pedal rate between 70 and 90 rpm, what was reproduced for subsequent tests. The power was then increased in a ramp fashion of 30 W/min until volitional exhaustion or till one of the American College of Sports Medicine established criteria for maximal testing was reached [17]. Verbal encouragement was given to the participants during the test. GET was determined by (1) the first disproportionate increase in carbon dioxide output (${\dot{\text{V}}\text{O}}_{2}$) from visual inspection of individual plots of ${\dot{\text{V}}\text{CO}}_{2}$ vs. ${\dot{\text{V}}\text{O}}_{2}$; (2) an increase in ventilatory equivalents for oxygen and not in carbon dioxide; and (3) an increase in end-tidal O_2_ tension with no fall in end-tidal CO_2_ tension. ${\dot{\text{V}}\text{O}}_{2}$_peak_ was determined as the highest value in a 30-s range recorded before the participant volitional exhaustion.

On subsequent visits, participants performed a various combinations of work-to-work transitions between moderate (M, 6 min at the 80% of the GET), heavy (H, 6 min at 20% of the difference in power between GET and the ${\dot{\text{V}}\text{O}}_{2}$_peak_) and severe (S, 6 min at 60% of the difference in power between GET and the ${\dot{\text{V}}\text{O}}_{2}$_peak_) intensity exercises. After 3 min at rest and 3-min “unloaded” baseline cycling, participants started one of the following four protocols: (1) M followed by S (M → S); (2) H followed by S (H → S); (3) M followed by H and by S (MH → S); (4) S followed by 3-min rest (S) followed by 3-min “unloaded” baseline and by S (SPost). Note that the last protocol provided data for S and SPost.

During the session, participants completed exercise twice separated by 1-h break in pseudo-randomized manner as each exercise was performed once at first place and once in second place.

${\dot{\text{V}}\text{O}}_{2}$, pulmonary gas exchange and ventilation were computed breath-by-breath. Prior to each test, the calorimeter and turbine were calibrated using ambient air and gases of known concentration (O_2_ = 14.01%, CO_2_ = 6.03%) and 3-L calibration Rudolf syringe (cortex, Leipzig, Germany), respectively.

The breath-by-breath ${\dot{\text{V}}\text{O}}_{2}$ data were initially examined to eliminate errant values caused by coughing, swallowing, etc., and values laying more than three 3 SDs from the local mean. Linear interpolation was used to provide second-by-second data, and for each individual, identical repetitions were time aligned to the start of exercise and the ensemble averaged. Mono-exponential equation was computed to isolate the primary component of the ${\dot{\text{V}}\text{O}}_{2}$ kinetics using the iterative method proposed by Rossiter et al. [18]

$${\dot{\text{V}}\text{O}}_{2} \left( t \right) = {\dot{\text{V}}\text{O}}_{2} {\text{baseline }} + \, A_{p} \left( {1 - { \exp }^{{{-} \, \left( {t - \, TDp} \right)/\tau p}} } \right)$$

where ${\dot{\text{V}}\text{O}}_{2} \left( t \right)$ is the time course of ${\dot{\text{V}}\text{O}}_{2}$, ${\dot{\text{V}}\text{O}}_{2}$baseline is the oxygen consumption at the beginning of exercise, A_p_ is the amplitude, TD_p_ is the time delay, and τ_p_ is the time constant of the primary phase, respectively. The first 20 s of the pulmonary ${\dot{\text{V}}\text{O}}_{2}$ signal was removed from analysis since it has been demonstrated that the cardiodynamic phase of the ${\dot{\text{V}}\text{O}}_{2}$ kinetics does not represent an increased muscle O_2_ consumption [19]. Identification of the end of the primary phase was made by criteria consideration as recommended by Rossiter et al. [20] and Murgartroyd et al. [21]. The magnitude of the slow component (As) was defined as the difference between the ${\dot{\text{V}}\text{O}}_{2}$ projected primary phase and the averaged amplitude from the last 30 s of the response (termed ${\dot{\text{V}}\text{O}}_{2}$end). The Gain Amplitude (GainAp) was defined as the increase in ${\dot{\text{V}}\text{O}}_{2}$ above baseline per unit increase in external work rate above baseline, ${\dot{\text{V}}\text{O}}_{2}$/∆WR. The Gain End (GainEnd) as the sum of the A_p_ and As per unit increase in external work rate above baseline (A_p_ + As/∆WR). The mean response time (MRT) was calculated as the sum of the Td_p_ and τ_p_.

**Fig. 1:**
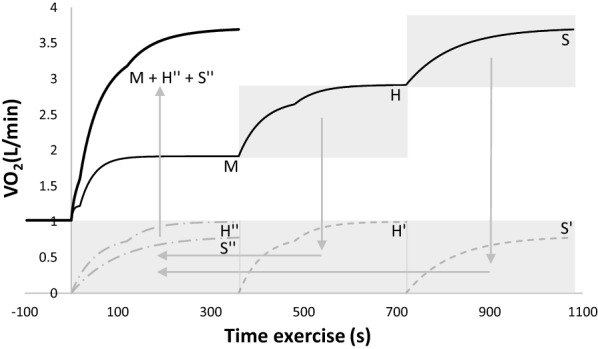
Illustration of reconstructed method used to analyze the work-to-work transitions protocol kinetics. Letters M, H and S represent the model for Moderate, Heavy and Severe intensities, respectively; H’ and S’ represent model for Heavy and Severe kinetic curves when ${\dot{\text{V}}\text{O}}_{ 2}$ baseline was set at zero; H” and S” represent heavy and severe kinetic curves when time was aligned to zero; M + H” + S” represents the reconstructed curve with the sum of the three different intensities

The superposition principle was applied to build the reconstructed curve. The start of each transition was time aligned and baseline was set at zero to sum the different kinetics curves as represented in Fig. 1. The parameters of the reconstructed curve were therefore calculated using the iterative method proposed by Rossiter et al. [18] (see above). Therefore, the reconstructed curve of M → S turns into MS; H → S turns into HS and MH → S turns into MHS.

### Data statistical analysis

Analyses were performed using Jamovi (Version 0.9.5.17 [Computer Software], retrieved from https://www.jamovi.org). Linear mixed model was the statistical test used to compare the ${\dot{\text{V}}\text{O}}_{2}$ kinetics parameters between the different conditions. Condition was the fixed effects and participant as the random effect. After inspecting residual plots, no obvious deviations from homoscedasticity or normality were present.

Linear mixed model was also used to temporally analyze data of the overall ${\dot{\text{V}}\text{O}}_{2}$ constructed kinetics, to compare the different conditions. The fit between the ${\dot{\text{V}}\text{O}}_{2}$ constructed kinetics was assessed by summing percent of time of ${\dot{\text{V}}\text{O}}_{2}$ constructed kinetics where differences were not significant. For all tests, the level of significance was set at 0.05 and dispersion about the mean expressed as SD.

## Results

Relative ${\dot{\text{V}}\text{O}}_{2 }$ was 55.77 ± 5.21 mL kg^−1^ min^−1^. The parameters of the ${\dot{\text{V}}\text{O}}_{2}$ response for each different transition are reported in Table 1. ${\dot{\text{V}}\text{O}}_{2}$baseline was significantly different (*p* < 0.001) between conditions except between S and Spost, and between H *→ *S and MH *→ *S conditions. Concerning Ap, only the comparison between H *→ *S or MH *→ *S was not significantly different. All other comparisons had a *p* value below 0.001, other than between S and Spost (*p* = 0.031). GainAp was significantly different between conditions (*p* < 0.003), other than between SPost and both S and M *→ *S. TDp was not significantly different between any of the conditions. As for ${\dot{\text{V}}\text{O}}_{2}$ baseline, τp was significantly different (*p* < 0.002) between conditions except between S and SPost, and between H *→ *S and MH *→ *S. Regarding MRT, H *→ *S and MH *→ *S were significantly slower (*p* < 0.001) compared with S, SPost, and M *→ *S, respectively. As was different between all conditions (*p* < 0.004), apart from between H *→ *S and MH *→ *S. Concerning ${\dot{\text{V}}\text{O}}_{2}$End, H *→ *S was significantly different to both M *→ *S (*p* = 0.007) and SPost (*p* = 0.037). GainEnd was not significantly different between S and SPost, and between H *→ *S and MH *→ *S conditions. All other comparisons were significantly different (*p* value range < 0.001 to 0.047).

**Table 1:** Comparison of the parameters of the ${\dot{\text{V}}\text{O}}_{2}$ response kinetics in the different transitions

	S	SPost	M → S	H → S	MH → S
$${\dot{\text{V}}\text{O}}_{2}$$ Baseline (L min^−1^)	1.07 ± 0.16	1.04 ± 0.21	1.85 ± 0.21^*§^	2.89 ± 0.23^*§£^	2.90 ± 0.25^*§£^
Ap (L min^−1^)	2.02 ± 0.27	2.16 ± 0.30^*^	1.43 ± 0.26^*§^	0.77 ± 0.15^*§£^	0.70 ± 0.15^*§£^
Gain Ap (mL min^−1^ W^−1^)	8.96 ± 0.73	9.56 ± 0.78	10.23 ± 0.60^*^	13.70 ± 1.50^*§£^	12.27 ± 1.16^*§£$^
TDp (s)	10.21 ± 5.54	11.76 ± 4.72	6.17 ± 5.37	6.06 ± 8.81	7.16 ± 9.83
τp (s)	29.31 ± 9.46	28.27 ± 6.79	46.51 ± 11.00^*§^	95.94 ± 19.61^*§£^	93.43 ± 13.83^*§£^
MRT (s)	39.52 ± 5.28	40.03 ± 4.21	52.67 ± 12.46	102.00 ± 27.57^*§£^	100.59 ± 18.17^*§£^
As (L min^−1^)	0.56 ± 0.13	0.44 ± 0.10^*^	0.33 ± 0.10^*§^	0.07 ± 0.07^*§£^	0.10 ± 0.07^*§£^
$${\dot{\text{V}}\text{O}}_{2}$$ End (L min^−1^)	3.65 ± 0.38	3.63 ± 0.35	3.61 ± 0.37	3.73 ± 0.33^§£^	3.70 ± 0.37
GainEnd (mL min^−1^ W^−1^)	11.47 ± 0.78	11.56 ± 0.67	12.62 ± 0.78^*§^	15.00 ± 1.96^*§£^	14.11 ± 1.55^*§£^

S: severe intensity; SPost: severe intensity after prior severe intensity; M: moderate intensity; H: heavy intensity. ${\dot{\text{V}}\text{O}}_{2}$ baseline: oxygen consumption at the beginning of exercise; Ap: amplitude of the primary phase; GainAp: increase in ${\dot{\text{V}}\text{O}}_{2}$ above baseline per unit increase in external work rate above baseline; TDp: time delay of the primary phase; τp: time constant of the primary phase; MRT: the sum of the TDp and τp; As: amplitude of the secondary phase; ${\dot{\text{V}}\text{O}}_{2}$ end, averaged amplitude from the last 30 s of the response; GainEnd: sum of the Ap and As per unit increase in external work rate above baseline. Values are presented as the mean SD

*Significant differences with S; ^§^significant differences with SPost; ^£^significant differences with M → S; ^$^significant differences with H → S (*p* < 0.05)

**Fig. 2:**
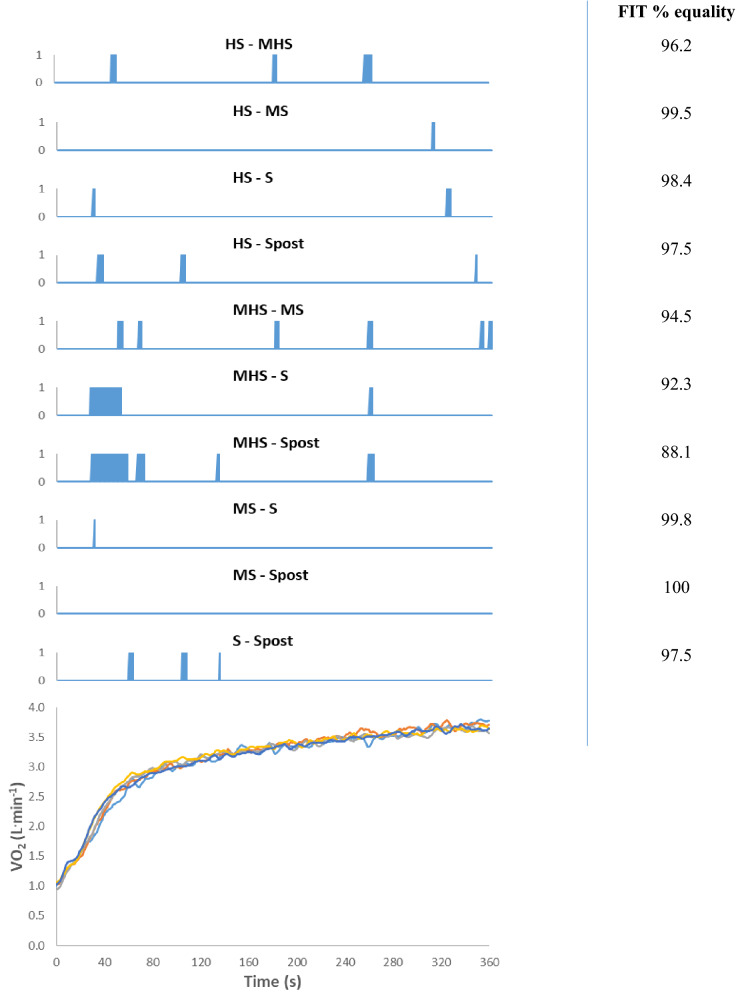
Pulmonary oxygen response (${\dot{\text{V}}\text{O}}_{2}$) of reconstructed curve. Upper panel illustrates time course comparison of the different reconstructed curves. Blue thick vertical squares represent the differences between protocols. On the right, fit calculations represent the percentage of equality between protocols. Lower panel shows the time course of all reconstructed curves. Light blue color represents MHS; red color represents HS; grey color represents MS; yellow color represents SPost; and dark blue color represents S. HS: reconstructed curve for Heavy and Severe intensities; MHS: reconstructed curve for moderate, heavy and severe intensities; MS: reconstructed curve for moderate and severe intensities; S, reconstructed for Severe intensity; SPost, reconstructed curve for severe intensity after prior severe intensity

The parameters of the results of the reconstructed method reported in Table 2 showed that ${\dot{\text{V}}\text{O}}_{2} {\text{baseline}}$, TDp, ${\dot{\text{V}}\text{O}}_{2}$End, and GainEnd were not significantly different between conditions. Ap was significantly different between S and both MS (*p* = 0.011) and MHS (*p* = 0.010). Furthermore, GainAp was significantly different between S and both MS (*p* = 0.007) and MHS (*p* = 0.007). Concerning τp, MHS was significantly slower compared with S (*p* = 0.011), SPost (p = 0.004), MS (*p* = 0.041), and HS (*p* = 0.038). Similarly, MRT from MHS was significantly slower compared with S (*p* < 0.001), SPost (*p* < 0.001), MS (*p* = 0.002), and HS (*p* = 0.016). Regarding As, S was slightly different (p = 0.043) from MS. Reconstructed kinetics of the different conditions are depicted at the bottom of Fig. 2. Fit calculation indicated similarity between the reconstructed curves; indeed, the similitude average was 96.38% with a maximum of 100% (MS vs. SPost). The largest differences were observed only during 11.94% of exercise duration (MHS vs. SPost), at the beginning of primary phase.

**Table 2:** Comparison of the parameters of the ${\dot{\text{V}}\text{O}}_{2}$ response kinetics of the reconstructed curve for the different transitions’ protocols

	S	SPost	MS	HS	MHS
$${\dot{\text{V}}\text{O}}_{2}$$ Baseline (L min^−1^)	1.07 ± 0.16	1.04 ± 0.21	0.97 ± 0.19	1.02 ± 0.23	1.01 ± 0.21
Ap (L min^−1^)	2.02 ± 0.27	2.16 ± 0.30	2.24 ± 0.29^*^	2.15 ± 0.30	2.24 ± 0.30^*^
Gain Ap (mL min^−1^ W^−1^)	8.96 ± 0.73	9.56 ± 0.78	9.95 ± 1.02^*^	9.55 ± 1.20	9.95 ± 1.12^*^
TDp (s)	10.21 ± 5.54	11.76 ± 4.72	11.41 ± 6.34	13.19 ± 4.73	10.64 ± 5.69
τp (s)	29.31 ± 9.46	28.27 ± 6.79	31.25 ± 7.51	31.00 ± 6.69	40.58 ± 11.68^*§£$^
MRT (s)	39.52 ± 5.28	40.03 ± 4.21	42.65 ± 5.86	44.19 ± 4.25	51.22 ± 7.07^*§£$^
As (L min^−1^)	0.56 ± 0.13	0.44 ± 0.10	0.41 ± 0.15^*^	0.53 ± 0.11	0.46 ± 0.17
$${\dot{\text{V}}\text{O}}_{2}$$ End (L min^−1^)	3.65 ± 0.38	3.63 ± 0.35	3.62 ± 0.41	3.70 ± 0.34	3.71 ± 0.38
Gain End (mL min^−1^ W^−1^)	11.47 ± 0.78	11.56 ± 0.67	11.77 ± 0.92	11.90 ± 1.20	11.99 ± 0.84

MS: kinetics sum of M + S; HS: Kinetics sum of H and S; MHS: kinetics sum of M + H+S. S: severe intensity; SPost: severe intensity after prior severe intensity; M: moderate intensity; H: heavy intensity. ${\dot{\text{V}}\text{O}}_{2}$ baseline: oxygen consumption at the beginning of exercise; Ap: amplitude of the primary phase; GainAp: increase in ${\dot{\text{V}}\text{O}}_{2}$ above baseline per unit increase in external work rate above baseline; TDp: time delay of the primary phase; τp: Time constant of the primary phase; MRT: the sum of the TDp and τp; As: amplitude of the secondary phase; ${\dot{\text{V}}\text{O}}_{2}$ end: averaged amplitude from the last 30 s of the response; GainEnd: sum of the Ap and As per unit increase in external work rate above baseline. Values are presented as the mean SD

*Significant differences with S; ^§^significant differences with SPost; ^£^significant differences with MS; ^$^significant differences with HS (*p* < 0.05)

## Discussion

The main finding was that the reconstructed ${\dot{\text{V}}\text{O}}_{2}$ kinetics, using a novel approach of combining Henneman’s principle with the principle of superposition, had a similar kinetic curve (96.4 ± 3.6% of similarity between conditions) to a simple transition at the same final severe intensity.

As hypothesized, when transitions started from a higher intensity, τ and Gain model parameters increased while amplitude parameters decreased, although ${\dot{\text{V}}\text{O}}_{2}$ end at the final transition was similar. These results are in line with previous studies [13, 22], which were interpreted as a reflection of metabolic differences in the pool of muscle fibers recruited under these specific circumstances. Indeed, the elevated baseline in the work-to-work protocols implies that type I MU are already recruited according to the well-established size principle of MU recruitment [23]; consequently, only a percentage fibers residing higher in the recruitment hierarchy would be activated during the second part of the protocol [13]. Type II muscle fibers are characterized by reduced mitochondrial content [24], lower oxidative enzyme activity [25] and greater ATP cost for force production [26]; therefore, slower ${\dot{\text{V}}\text{O}}_{2}$ kinetics and lower efficiency [27]. Consistent with the increased contribution of fibers with lower oxidative efficiency, the Gain amplitude of the primary phase was progressively increased when exercise was initiated from an elevated baseline [13, 22].

The second hypothesis was also validated since there were only scarce differences between the reconstructed kinetics and the work-to-work transitions (Fig. 2). The disparities were mainly in the first 40 s of the exercise due to a significant slower τp in MHS compared with the other conditions. However, during the time course of ${\dot{\text{V}}\text{O}}_{2}$_SC_ and at the end of exercise, only sporadic differences were observed.

The fact that each severe exercise, preceded by a different modality producing a different fatigue, had similar reconstructed kinetics, suggesting that (a) fatigue was not the main process involved in the ${\dot{\text{V}}\text{O}}_{2}$_SC_; (b) the progressive fiber recruitment, due to fatigue, was consequently not required for the development of the ${\dot{\text{V}}\text{O}}_{2}$_SC_. During work-to-work exercise, new fibers are activated at the beginning of each transition, modeling the ${\dot{\text{V}}\text{O}}_{2}$ kinetics response. The result of temporally aligning the kinetics of ${\dot{\text{V}}\text{O}}_{2}$ at the beginning of each transition and summing them seems to be similar to the result of a complete stimulation of the different fibers involved in a single transition of severe intensity exercise. This is consistent with the fact that the kinetics shape is mainly driven by the metabolic response profiles of different fibers populations. Several studies have demonstrated the link between the different profiles of fibers and the appearance of the ${\dot{\text{V}}\text{O}}_{2}$_SC_. The first authors to demonstrate that type I muscle fibers were significantly correlated with the ${\dot{\text{V}}\text{O}}_{2}$_SC_ were Barstow and colleagues [28]. They exercised participants at ∆50% and took muscle biopsies of the vastus lateralis for determination of fiber type. Participants with a higher percentage of type I muscle fibers had a higher primary phase and this was significantly correlated with the amplitude of the ${\dot{\text{V}}\text{O}}_{2}$_SC_ (*r* = − 0.83). Previous findings indicate significant correlations between the percentage of type II muscle fibers and markers of aerobic fitness and the relative magnitude of the ${\dot{\text{V}}\text{O}}_{2}$_SC_ (*r* = 0.60; *p* < 0.01) and (*r* = −0.73; *p* < 0.01), respectively [29]. These findings are in line with other studies relating to the percentage of type I muscle fibers with an improved efficiency, or reduced ${\dot{\text{V}}\text{O}}_{2}$, for the same work rate in cycling [30] or running [31]. Pringle et al. [32] took muscle biopsies from 14 participants for histochemical determination and made them complete square-wave cycling tests at moderate, heavy and severe intensities. Percent of type I muscle fibers was correlated with the amplitude of the ${\dot{\text{V}}\text{O}}_{2}$_SC_ for heavy (*r* = − 0.74; *p* < 0.01) and severe (*r* = − 64; *p* < 0.05) exercises and with τ of the primary component (*r* = − 68; *p* < 0.01) in heavy intensity. Indeed, after a protocol aiming for the depletion of glycogen from type II muscle fibers, there was a decrease in the amplitude of the ${\dot{\text{V}}\text{O}}_{2}$_SC_ [33]. Deley et al. [34] showed that after pre-fatiguing type II muscle fibers, the amplitude of the ${\dot{\text{V}}\text{O}}_{2}$_SC_ was significantly reduced, concluding that the recruitment of type II may be involved in the ${\dot{\text{V}}\text{O}}_{2}$_SC_ phenomenon. Krustrup et al. [35] confirmed the idea that the energy turnover and ATP cost were higher for type II fibers when a neuromuscular blockage of type I was performed. Certainly, muscle O_2_ uptake was 20% higher and MRT was longer in type II muscle fibers, supporting the idea that type II fibers had slower kinetics and greater ATP cost than type I during dynamic exercise.

Finally, if the metabolic characteristics of the different fibers shape the ${\dot{\text{V}}\text{O}}_{2}$ kinetic curve and fatigue does not play a role in the development of ${\dot{\text{V}}\text{O}}_{2}$_SC_, neither will the progressive recruitment of these fibers. This result has been seen in isolated gastrocnemius dogs [36] and in the vastus lateralis in humans [37] when all muscle fibers were activated or when a systematic increase in the cost of O_2_ per unit of external power was concomitant with no changes in iEMG, respectively. Taken together, these results suggest a lack of progressive muscle fiber recruitment during ${\dot{\text{V}}\text{O}}_{2}$_SC_.

## Conclusion

These results confirm that τ is significantly smaller between low work rate transitions compared with transitions between high work rate intensities. In addition, the ${\dot{\text{V}}\text{O}}_{2}$ severe intensity kinetic curve is similar to the reconstructed kinetics curve resulting from combining Henneman’s and superposition principles. These findings are consistent with the appearance of the ${\dot{\text{V}}\text{O}}_{{2{\text{sc}}}}$ and maybe linked to the intrinsic differences in metabolic properties of different fiber types.

## Data Availability

The datasets used and analyzed during the current study are available from the corresponding author on reasonable request.
